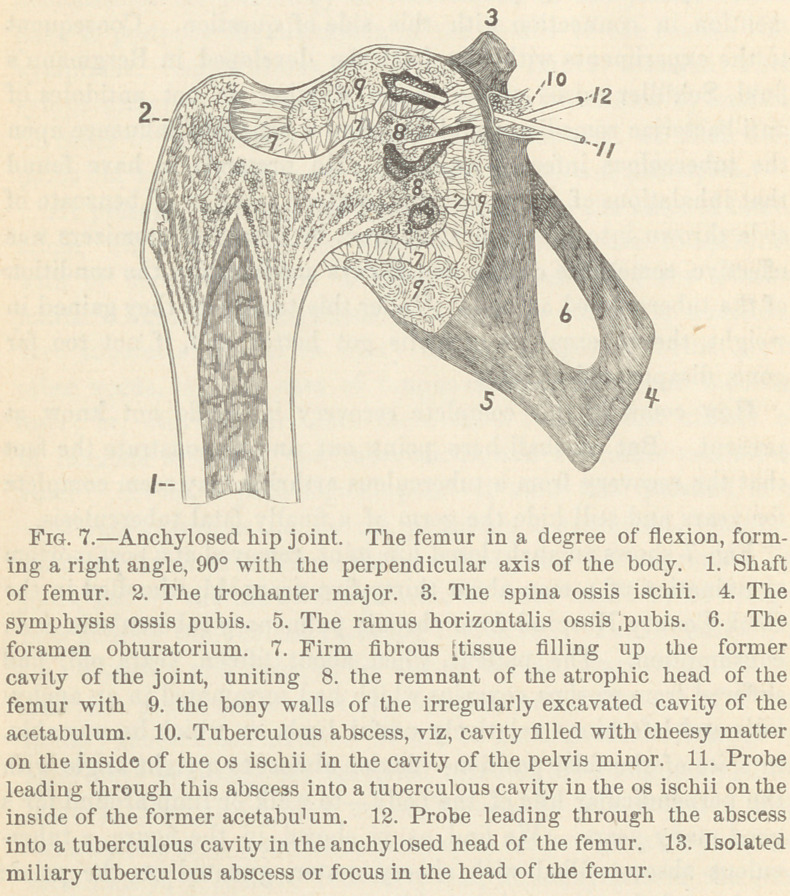# Select Topics of Modern Surgery

**Published:** 1880-05

**Authors:** 


### Original Communications.

---

ARTICLE III.

SELECT TOPICS OF MODERN SURGERY. Illustrated by Cases from the Hospital Service and Private Practice of DRS. E. W. LEE and CHR. FENGER. Read before the West Chicago Medical Society.

TUBERCULOSIS OF JOINTS.

Miliary tubercles in the synovial membranes of the joints were first mentioned by the father of modern pathology—Rokitansky. (Pathological Anatomy, Sydenham Society Edition, London, 1850. Vol. III, p. 296.) No attention was paid to the subject, however, for a number of years.

Richard Volkmann, the eminent author in modern surgery on bones and joints, was the first surgeon who published in the leading surgical literature investigations which confirmed Rokitansky's previous observations on tuberculosis of the bones and joints. (Pitha and Billroth, Chirurgie, die Krankheiten der bewegungs Organe. Abschitt V, p. 260.) Volkmann considered tuberculosis of the bones and joints as a rare disease, as is readily seen in his description of the white swelling and the caries of the adjacent bones. He justly pointed out the errors of the previous French authors on the subject, Nélaton and Lebert, who, retaining the original and old doctrine of Laennec, "where cheesy matter is found tubercles pre-existed," had described tuberculosis of bones and joints where no miliary tuberculosis had been demonstrated. Laennec's mistake as to the identity of cheesy matter and tubercle was cleared up by Virchow, who proved that necrosis of any variety of original or newly-formed tissue might result in its transformation into cheesy matter, and that consequently it was premature to conclude that because we found cheesy matter present, it was due to the presence of previously existing tubercle.

Thus utilizing Virchow's observations, Volkmann was right in repudiating Nélaton's and Lebert's descriptions as false and unfounded, and he was at that time justified in pronouncing it an original discovery, the tuberculosis of the organs in question.

We were not able to make an indisputable diagnosis of miliary tubercle until Langhans, about ten years ago, gave an exact histological description of the young growth in its most minute details. Previously Virchow's description was accepted, viz., that it consisted of a small conglomeration of round lymphoid cells imbedded in a fine stroma of non-vascular connective tissue; and that the fate of these cells was a speedy fatty degeneration, due to lack of blood vessels in the little growth. By the microscopic examination alone we could not make a differential diagnosis between miliary tubercle and the miliary forms of malignant growths (as carcinoma and sarcoma). Also the same difficulty presented itself in the microscopic examination of normal elements, such as the lymphatic follicles of the intestinal tract; the tonsils; solitary follicles from Peyer's patches and from the colon, in which a diagnosis based upon histological grounds could not be made from miliary tubercle. Since Langhan's investigations we have been able to recognize, by aid of the microscope, the miliary tubercle even in places and tissues where none of its well-known characters were visible to the naked eye to call our attention to the true nature of the disease.

The unmistakable anatomical characters of the miliary tubercle thus established enabled Köster (*Ueber fungöse Gelenkentzündung. Virchow's Archiv*, 48, *p.* 49) to make the most remarkable and unexpected discovery that, in the great majority of the cases of so-called white swelling, tumor albus, caries of joints, chronic destructive inflammation of joints, miliary tubercles were found to be the origin of the disease. Thus the same miliary tuberculosis that in the lungs, brain, and uro-genitary organs was recognized as an inevitably fatal disease of variable duration, made its appearance in the joints in a disease, the prognosis of which as to the life of the patient was not considered grave, provided the proper treatment was resorted to.

It was quite natural that Köster did not believe the facts evident to his own eyes, and expressed the opinion that fungous arthritis (white swelling) in spite of the thousands of miliary tubercles so often found in the various tissues of the affected joint, was a separate disease from the true tuberculosis of the joints, producing or accompanying the fatal and general tuberculosis of the internal organs.

It was now necessary for surgical pathology to engage in the investigation of a question of such vital import, and to sift our knowledge of tuberculosis in general, and specially with reference to its bearing on, and the consequent treatment of this disease in the joints.

The latest investigators, Volkmann, Friedländer, Schüppel, König and ourselves, though not numerous, form but one conclusion, and the results of their investigations all tend to confirm the true tuberculous character of the disease in question and do not admit the distinction of Köster between general tuberculosis and local tuberculosis of joints—the one benignant, the other fatal—but rather seem to foreshadow a change in our inherited views of the necessarily fatal prognosis of every disease originating in, or complicated with the presence of miliary tubercle in the affected tissues.

Numerous future investigations will be required to determine the theoretical as well as the practical importance of the new epoch in the doctrines of tuberculosis. Hoping that surgeons in this country will take their part in the solution of these problems, we will give the pathological anatomy of the disease, and later point out the main question in its relations to general tuberculosis, and its consequent rational treatment as far as our actual standpoint will permit.

The miliary tubercle shown in Fig. 1 is a small round tumor not visible to the naked eye. Its main characteristics are as follows: I. The giant cell reticulum—forming the central part of the growth and containing one or more giant cells. II. The lymphoid reticulum containing a large number of lymphoid cells, forming the peripheral part of the tubercle and surrounding the former.

The giant cell is a large, irregular and uniformly finely granulated protoplasmic mass, *a*, Fig. 1, containing a variable number of large oval nuclei, with one or more nucleoli. The nuclei are either scattered irregularly over the cell mass or are arranged in a row along the peripheral part of the cell. From the surface of the giant cell pass out long branched processes, which are continuous with the reticulum of the central portion of the tubercle.

The giant cell reticulum, Fig. 1. *b*, forms a net-work with large round or irregular meshes. Many of the meshes are empty, i. e., do not contain any cells, but are filled up with clear serous fluid. In a few of the meshes are found large epithelial cells with a plainly visible protoplasmic surrounding, and a well defined round or oval nucleus. A large number of the meshes contain two, three or more small lymphoid cells or nuclei, many of which are highly refracting.

Outside of this giant cell reticulum we find the peripheral part of the tubercle consisting of the (*c*) lymphoid reticulum or small celled tissue of adenoid-like structure. The meshes of this reticulum are narrow, long, oval, or spindle-shaped spaces, their long diameter being perpendicular to the radius from the center of the tumor. They are filled entirely with innumerable small, round more or less refracting cells or nuclei.

The surrounding tissue, of recent growth in which the tubercles are imbedded, has, in its young state, invariably all the characteristics of the so-called adenoid or lymphoid structure, (as is shown in Fig. 2.) It derives its name from its similarity in structure to the lymph-glands, or the tonsils, or the adenoid tissue as we find it along the whole of the intestinal tract, or the adenoid vegtations from the naso-pharyngeal cavity. It consists of a fine connective tissue net-work with round or oval meshes, filled with lymphoid cells and nuclei.

It contains numerous blood vessels with thin walls, the external coats of which are transformed into or take part in the formation of the branches of the reticulum. This adenoid-like structure of the connective tissue around the tubercles we found in all cases. [The same structure may be found in young connective tissue, the formation of which plays no part in the growth of tubercle.] It grows out between the bundles of the normal tissues—fibrous, muscular, etc.—creeps along the vessels into the fatty tissue and causes thus the thickening of the soft structures of the joints, as we so often find it in the white swelling, where the capsule is transformed into a grayish, white, firm, inelastic, fibrous mass, varying from one to two lines to half an inch or more in thickness.

A transverse section through such a thickened capsule is shown in Fig. 3.

Fig. 3.—Thickened capsule of knee joint; case of resection of Dr. Fenger: *a*, Outer layer of normal fibrous tissue; *b*, Bundles of fibrous tissue inlaid with lymphoid tissue; *c*, Inner layer of adenoid tissue containing numerous tubercles; *d*, Tubercles without giant cells; *e*, Tubercles with giant cells; *f*, Vessels.

The following are the microscopic features: *a*, An outer layer of normal fibrillar connective tissue of the fibrous capsule of the knee joint. Inside of this, at *b*, two obliquely-cut bundles of fibrous tissue, partially transformed into adenomatoid tissue. Finally the thickest inner layer, *c*, consists of adenomatoid tissue, with a large number of disseminated miliary tubercles, some of which contain a large giant cell, *e*, *e*, while others, *d*, *d*, *d*, have a center consisting of the giant cell reticulum without giant cells, but all of them are surrounded by a darker ring of the lymphoid reticulum.

The metamorphoses and alternate fate of the miliary tubercle, as we learn from the most recent authors, are as follows: After an, as yet, undetermined term of existence, either simple atrophy or fatty degeneration commences in the center of the tubercle, where first the cellular elements and later the interstitial tissue become trasformed, partly into irregularly-shaped, soft corpuscles—the so-called tubercular corpuscles—and partly into a finely granulated fatty detritus.

In rare instances simple atrophy may occur in the cellular elements alone, meanwhile the reticulum gets thickened and the tubercle becomes transformed into a hard, horny mass. This transformation is considered a cornification, and is only rarely observed.

The partially atrophied and fatty degenerated tubercle will further undergo one of the three following metamorphoses:

Resorption may take place, especially where the tubercles are widespread and isolated. Wagner (Manual of General Pathology; translation; New York, 1876) regards this as a most rare occurrence.

Calcification is more frequent, and means transformation of the fatty detritus into cheesy matter, intermixed with chalky masses, finally encapsulated, viz.: surrounded by a capsule of dense cicatricial connective tissue.

Softening and liquefaction is justly considered the most important change, because it is most often accompanied by suppurative inflammation in the surrounding tissue. If the seat of the turbercle is on the surface—mucus membrane, skin, etc.—ulcers are formed, and if they are located in the interior of the organs, tuberculous cavities result, and tuberculous abcesses form and often become the starting point for suppurative processes, *i. e.*, the formation of abcesses around the tuberculous foci.

Wagner only expresses the general opinion of the profession when he says that the last named form of fatal transformation of the tubercles is of most frequent occurrence. In the remainder of this paper it will be seen that we do not quite agree with him in this conclusion. We consider, and are obliged to state, that as far as the tuberculosis of joints is concerned, absorption of the tubercles is of frequent occurrence.

### A.—PRIMARY OSTEO-TUBERCULOSIS IN THE EPIPHYSES NEAR THE ARTICULAR SURFACE OF THE BONES.

The tuberculosis of the joints originates, according to Kocher and Volkmann, in the great majority of cases, not in the capsule or any other of the soft parts, but in the spongy structure of the epiphyseal extremities constituting the joint, *i. e.*, it commences outside the joint as a tuberculous caries, or rather as a local miliary tuberculosis of the epiphyses.

A typical specimen illustrating this fact is shown in Fig. 4.

It represents a longitudinal cut through the knee-joint removed at a post-mortem examination of a patient at the Cook County Hospital.

A short résumé of the records gives the following facts taken from the history published by Dr. Murphy, of Cook County Hospital, in the *Chicago Medical Gazette :*

" *Primary osteo-tuberculosis of the superior extremity of the*

*right tibia, tuberculous arthritis of right knee-joint. Secondary tuberculosis of inguinal glands, bladder, ureters, right kidney, and peritoneal cavity, commencing chronic tuberculosis of lungs, "pleuritis pyæmica."*

Married woman æt. twenty; family history good as to tuberculosis; an aunt died of cancer of the mamma. Five years ago she began to have some dull pain in the right knee-joint; there was occasionally some swelling of the joint, which would last but a few days and then entirely subside, but the pain was a constant annoyance and seemed to bear no relation to the swelling of the joint. The pain was exaggerated by long standing or walking, but sudden pressure caused her no inconvenience whatever.

Eight months ago, just previous to urinary trouble from the commencing tuberculosis of the bladder, the parts about the joint became much more swollen, the knee was flexed at a right angle from which position motion caused severe pain. It thus continued very bad for about two months and then subsided gradually, but remained flexed, though to a less degree. There was no anchylosis for it allowed quite a latitude of motion. On moving her from her home to the hospital the knee was considerably shaken, which caused an acute swelling of the soft parts. On admission the right knee was found flexed at a right angle, from which position she could only move it to a slight degree. The entire articulation is considerably swollen and tender. The swelling appears to be partly due to a distension of the synovial sac, but also to a general infiltration of the soft tissues of the joint.

*There are no sinuses leading into the joint and no scars resulting from previovs abscesses.*

For the remainder of the history we shall only state that for the last eight months she had suffered from urinary trouble and pains in the hypogastrium, signs of a chronic, steadily progressing cystitis and pelvic cellulitis, accompanied by loss of strength, anæmia, and latterly by fever.

The diagnosis was considered to be a tuberculosis of the organs before mentioned; the prognosis fatal, and death resulted twenty days after admission. The post mortem examination revealed the following:

The right lung contains numerous nodules consisting of peribronchitic granules; in the lower lobe a sub-pleural abscess about the size of an acorn containing sanious pus. The left lung contains peribronchitic granules but no abscesses.

The entire wall of the peritoneal cavity is studded with miliary tubercles; the mesenteric glands greatly enlarged and mostly degenerated into cheesy matter.

*The inguinal glands on the right side enlarged, softened and broken down into cheesy abscesses.*

In the retro-peritoneal tissue above the right kidney is a large abscess filled with fluid pus, the walls of which are irregular, ulcerated, and contain numerous miliary tubercles.

The right kidney is filled with miliary and conglomerated tubercles which in some places are degenerated into large cheesy masses. In the pelvis of the kidney, the walls of the enlarged ureter and the bladder, are found large confluent tuberculous ulcers leaving only small islands of recognizable mucous membrane on their walls. The connective tissue in the pelvis minor is infiltrated with tubercles and cheesy matter, in the midst of which are found two tubercular abscesses, one of which opens into the bladder, and the other into the vagina.

The right knee joint is bent almost at right angles, and a little motion is obtainable. The circumference of the joint is somewhat enlarged. The skin covering it is natural. There are no sinuses nor cicatrices and no abcesses around the joint.

The cavity of the joint is filled with whitish, dry, cheesy matter of the consistence of putty. After the removal of this matter we find the capsule a little thickened and rigid, its inner surface being slightly uneven and velvety, but no miliary tubercles visible.

The cartilaginous covering of the articular ends of the bones is mostly destroyed and only small, irregular islands of cartilage left on the denuded and roughened osseous surfaces. In the head of the tibia, close to the joint, is seen a cavity (10) occupying the spongy portion and measuring 1.5 centimeters, in antero-posterior diameter 1 centimeter from above down and 2 centimeters laterally. This cavity is lined with a soft, grayish membrane 1 to 2 millimeters in thickness. This membrane consists of adenoid tissue, in which are imbedded thousands of miliary tubercles.

The cavity is filled with whitish, cheesy matter, similar to the cheesy matter found within the joint. Microscopically, this cheesy matter is seen to consist of a finely-granulated, fatty mass, with no recognizable cellular elements in it. This tuberculous cavity is in communication with the joint in two places; the one at its upper posterior part, as shown in the figure; the other at the right part of the roof, through the external articular surface of the tibia. Near the upper posterior margin of the tibia (at 11) is a small, round cavity of tubercular origin near the surface of the bone, but not as yet communicating with the joint. In the external condyle of the femur (12) we find a deep transverse, loss of substance or superficial erosion or carious ulcer, measuring 3 centimeters in length, 1 centimeter in width, and 4–5 milimeters in depth. Its walls are irregular and here and there are variable sized sequestra.

The local osteo-tuberculosis in the epiphyses, as illustrated in the above case, is, according to R. Volkmann, the eminent surgeon and pathologist and author of an excellent monograph on the diseases in question,* in the great majority of cases, the starting point of the chronic fungous arthritides, the scrofulous arthritides, the white swelling of the joints.

The tuberculous osteitis or osteo-myelitis in the epiphyses may be circumscribed or diffuse. Most generally it is local.

Through cheesy degeneration of the tubercles, that fill the place of the absorbed osseous tissue, there is formed a cavity filled with cheesy matter and lined with a membrane composed of living adenoid tissue and tubercles. In a number of cases there will be found variable sized pieces of dead bone or sequestra in those cavities for the following reasons: *First*, the tuberculous osteo-myelitis cuts off an island of unabsorbed osseous tissue from its nutritive supply and finally isolates it as a loose sequestrum. *Second*, a sudden cheesy degeneration and consequent death takes place in part of the tubercular, infiltrated, spongy osseous tissue before the absorption of the osseous substance can take place. The portion thus affected must therefore at a later

*Sammlung Klinischer Vorträge, No. 168–169, 1879. Ueber den Character und die Bedeutung der fungösen Gelenkentzündungen.

period, form a loose sequestrum surrounded by cheesy matter, and the living tuberculo-adenoid membrane.

The cancellous structure surrounding the tuberculous cavity is in most cases healthy, the miliary tubercles not spreading far into the medullary spaces, but rather keep together in one circumscribed tumor as we find it in the conglomerated tubercles of the brain where the thin grayish membrane of miliary tubercles that circumscribes the central yellow cheesy mass is surrounded by healthy brain tissue.

This local characteristic of the tuberculosis is a fortunate peculiarity inasmuch as it enables us to remove the whole of the diseased part. It will be sufficient to empty the cavity and scrape out its lining membrane with the curette, or sharp spoon, or concave chisel, or gouge, for the complete eradication of the tubercles of the affected locality. We are thus often able to stop the progress of the disease with a relatively insignificant loss of substance of the epiphysis in question. Those primary local tuberculous foci are in some cases single, in others multiple, but the number is generally limited. Usually only one of the bones of the joint is the seat of them but they may be found simultaneously in two or all of the bones constituting the joint. Their place of election is close to the articular surface, immediately beneath the articular cartilage, and in children, in the neighborhood of the epiphyseal cartilage. A small subchondral primary tubercular focus may disappear in the later destruction of the joint, and thus we are unable to trace, at operation or autopsy, the tuberculous arthritis to its very origin.

There are found, however, and fortunately rarely, cases where the tuberculosis is not thus localized, but where the eruption of miliary tubercles spreads diffusely over the epiphyses resulting in an equally diffuse cheesy degeneration. In such cases the removal of the bone must be much more extensive, thus increasing the danger of operative interferance. Volkmann's prognosis in these cases is grave as regards the danger of general tuberculosis, though not necessarily fatal.

In another class of cases, still more rare, a diffuse caseous degeneration takes place throughout the whole of the epiphysis where one or more local tuberclous foci are seated, and on section of the spongy osseous tissue we find it yellow, dry and bloodless from cheesy degeneration, and dying as a result of the diffuse osteo-myelitis of the epiphysis. In these cases removal of the entire bone is demanded.

The local tuberculosis or cavity will slowly grow larger and larger, till finally it will perforate to the surface of the bone. Generally the disease is not revealed until this event occurs. Symptoms during the stage of development of the cavity but previous to its perforation, are conspicuous by their absence. There is, as a rule, no pain or disturbance whatever; but as soon as the surface of the bone is reached the adjoining organs or super-imposed tissues become poisoned by contact with the cheesy matter and inflammation results. Naturally we might infer that it is a matter of great importance whether the cavity opens within the joint or reaches the bony surface outside the same, and would modify greatly our prognosis as to the result of the disease and the fate of the patient.

If the tuberculous cavity opens outside the joint, the integrity of the latter will remain unimpaired, and extra-articular abscesses will form. Sooner or later these abscesses will reach the surface of the part by natural processes or be evacuated by surgical aid. They contain either normal pus or a thin, slimy or scrofulous pus.

The wall of the abscess is lined with a grayish, loosely-adherent membrane, and in structure it consists of adenoid tissue, studded with miliary and conglomerated tubercles, often visible to the naked eye as small, round, grayish, white nodules, similar to the tuberculosis of the large serous cavities—peritoneum and pleura —with which we are all familiar.

The fistulous sinuses resulting from these abscesses have little tendency to close up, partly because they communicate with the tuberculous cavity in the bone, and partly on account of the tubercles lining their walls.

The diagnosis of the tuberculous character of the disease can readily be determined by the removal of a portion of the wall by aid of a curette and examination of the tissue under the microscope.

At this stage of the peri-articular tuberculosis, proper treatment will, in some cases, arrest further progress of the disease and save the endangered joint. The plan of the operation is obvious. We must cut through the fistulous tract down to the surface of the bone, dilate the osseous opening with chisel or gouge, lay open the tuberculous cavity, remove its contained cheesy matter, and sequestra, and scrape or dig away the whole of the tuberculous, infiltrated tissues.

Cases in which the neighboring joint has been saved have been reported by Kocher after the foregoing operation was performed. (Zur Prophylaxis der fungösen Gelenkentzündnng—[Volkmann Klinische Vorträge, No. 102.)

### B.—THE TUBERCULOUS ARTHRITIS.

I.—*The Consecutive or Secondary Tuberculous Synovitis.*

When the osteo-tuberculous cavity opens into and empties part of its cheesy, infectious contents into the joint, a tuberculous inflammation results. In the great majority of cases this arthritis assumes, from the very beginning, the same chronic character as did the tuberculous caries of the epiphysis. This chronic, slowly-developing fungus arthritis has been termed pannous arthritis, from its anatomical resemblance to pannous inflammations of the cornea. The synovial membrane becomes injected and swollen, the articular cartilages replaced by vascularized granulations or connective tissue. In the cavity of the joint may be found an augmented amount of synovial fluid, somewhat whitish from admixture with lymphoid elements. Slight pain, impaired motion, moderate swelling, no palpable signs of effusion within the joint, and sometimes slight, muscular contractions are the symptoms met with in this condition.

This pannous arthritis may set in before caseous matter is effused into the joint (Volkmann and Kocher) from irritation produced by the tuberculous inflammation in such close proximity to the articular surfaces. As the pannous arthritis has a tendency to obliterate the cavity of the joint and to terminate in a more or less complete false anchylosis, it often happens, therefore, that the caseous matter opens into an already partially obliterated synovial cavity. If this be the case the reactionary inflammation will be far less vehement than if the cheesy matter entered a normal-sized and healthy synovial cavity.

The foregoing reasons account for the well-known fact that the fungous arthritis sometimes sets in with the symptoms of an acute inflammation; generally, however, it has a chronic character from the start. This chronicity may be interrupted by acute but transient exacerbations, to be accounted for by the cheesy matter as above stated.

The most serious consequence of this tuberculous arthritis is its destructive influence on the integrity of the affected joint. The articular cartilage or the periosteum disappears or is transformed into miliary tuberculous adenoid tissue, and the epiphyseal arthritis of the bones becomes destroyed by a carious tuberculous osteitis, extending from the surface to the deeper tissues of the bone.

The secondary tuberculous destruction of the epiphysis is sometimes uniformally distributed over the entire articular surface, whilst in other cases it is localized and forms shallow excavations. A specimen representing the latter condition is shown in Fig. 5.

FIG. 5.—Upper extremity of the right femur removed by excision by Dr. E. W. Lee, from a six year old girl suffering from morbus coxarius with a large anterior abscess. (The details of this successful operation will be published in another paper.) 1. The great trochanter. 2. The lesser trochanter. 3. The neck of the fumur. 4. The head of the femur reduced to one-third its normal size, with irregular circumference and denuded roughened surface. 5. Tuberculous cavity in the head and neck, partially filled with 6. A mass of reddish-gray soft tissue—fungoid granulations—consisting of adenoid tissue with miliary tubercles.

This upper extremity of the right femur removed by excision by Dr. E. W. Lee, shows, besides considerable superficial destruction of the head, (4) a shallow cavity (5) two centimeters long, one and a half centimeters broad, and half a centimeter in depth, situated in the inferior portion of the neck and also in the head of the bone. This cavity is mainly filled with a mass of reddish-gray soft tissue—(6) fungous granulations—in which fine yellow specks or points are seen, but no gray miliary nodules are visible to the naked eye. The miscroscopic examination of this tissue shows it to consist of adenoid tissue and miliary tubercles.

In this secondary, diffuse and superficial tuberculosis of the bones—tuberculous periostitis, osteitis and caries—the osseous tissue is transformed into masses of reddish-gray soft tissue, viz., fungous granulations—hence we find in this tissue, situated between the adenoid tissue and the tubercles, spiculæ of bone undergoing the process of absorption.

FIG. 6.—Miliary tuberculous fungous granulations from elbow joint. Excision by Dr. Lee for chronic fungous arthritis with impending anchylosis. No periarticular abscesses nor fistulæ. Old primary osteo-tuberculous cavity in the head of the radius. Secondary superficial tuberculosis of the olecranon and the lower extremity of the humerus. 1. Young connective tissue of mainly adenoid character, with 2. Smaller and larger lymph spaces. 3. Half of miliary tubercle with the 4. Giant cell in the center. 5. Spiculum of osseous tissue.

In Fig. 6 is shown a section of fungous granulations covering the atrophied anterior surface of the lower extremity of the humerus. This was removed by excision of the elbow joint in a case of chronic fungous arthritis in Dr. Lee's practice. We find a young connective tissue, rich in cells and mainly adenoid in character (1) containing a number of variable-sized round empty spaces, with serous fluid filling the large lymph spaces. 2, 2, 2. At 3 is a miliary tubercle with a giant cell (4) in its center, and at 5 is an irregular island of osseous tissue (spiculum) with bone corpuscles surrounded partly by lymph spaces and partly by adenoid tissue.

This little spiculum of osseous tissue is part of the old bone in the stage of absorption, because in no part of its surface is found either osteoblasts or connective tissue—corpuscles or cells undergoing the process of transformation into bone corpuscles.

The effect of cheesy matter from a tuberculous cavity upon the healthy synovial cavity has been demonstrated by the experiments of Prof. Hüter* of Greifswald.

He made an emulsion of tuberculous sputa from consumptive patients, and then injected one-third to three-quarters of the contents of a common subcutaneous syringe into the cavities of the knee and ankle joints of five dogs. After eight to fourteen days swelling commenced in the joint, abscesses reached the surface and left sinuous openings, discharging a sero-purulent fluid. The animals became emaciated, and diarrhæa set in. One of the dogs died of acute general miliary tuberculosis. When the inflammation of the joint subsided, the general condition of the animals improved, he also noticed. In the joints of the animals which he killed was constantly found a condition of fungous arthritis in different stages. The fungous granulations of such a joint were emulsionized with water and injected into the peritoneal cavity in two healthy dogs. Four weeks later the animals

* Die Experimentelle Erzeugung der Synovitis granulosa an Hunde, und die Beziehungen dieser Gelenkentzündung zur Tuberculose, Deutsche Zeitschrift für Chirurgie Bd. XI Heft 3 und 4, Centralblatt für Chirurgie, No. 43, 1879.

were killed, and he found swelling of the retro-peritoneal glands, desquamative pneumonia in the lungs—consumption—miliary tubercles in the diaphragm and the pleural cavities. He therefore concluded that the fungous arthritis—the synovitis granulosa—is a tuberculous disease.

II. THE PRIMARY TUBERCULOUS SYNOVITIS.

A priori there is no reason why the tuberculosis should not commence in the synovial membrane of a joint as well as in the adjoining bones. But a priori theories or reasonings are of little account when compared with observations based upon facts. The numerous authentic cases upon which Volkmann founded his monograph made him enunciate the opinion that the synovial tuberculosis in the great majority of cases is secondary—caused by a communication being established between the primary osteo-tuberculous cavity and the joint. We do not doubt but that his statement is in strict accord with the facts observed by him.

Volkmann admits that primary synovial tuberculosis is a rare event, and thus far we agree with him; but when he further states that primary synovial tuberculosis occurs rarely save in adults, and calls for a much more serious prognosis than the common secondary tuberculous arthritis, we beg leave to differ with him.

In one of our cases, where, in tuberculosis of the knee-joint, excision was performed by Dr. Fenger, there was no primary osteo-tubercular focus to be found in any part of the bones.

In the hip-joint represented in Fig. 5, we feel inclined to regard the tuberculosis of the neck and head, of the femur as secondary on account of the shallowness of the cavity, but we admit that our opinion here might be disputed. An important step toward the solution of this problem has been made by the recent experiments of Shüller (Centralblatt für Chirurgie, 1878, No. 43 and 1879, No. 19, also Archiv. für Experimenttelle, Pathologie and Pharmakologie, Bd. 11, Heft. 1 and 2, 1879; also Allgemeine medicinische Zentralzeitung, No. 82, 83, 84, 1879.)

The author being aware of the clinical fact that a slight trauma was often mentioned as a common factor in the etiology of the scrofulous, viz., tuberculous inflammations of the joints, desired to settle the following queries: Why can such an unimportant traumatism—in children, almost of daily occurrence—produce, in a very limited number of individuals, such serious consequences; and what are the predisposing morbid conditions in the organism of the individual subject to such grave results? Clinical observations have long since pointed out the coincidence, to say the least, between fungous arthritis, scrofulosis and tuberculosis. Villemin had produced tuberculosis by inoculation, Tappeiner by inhalation of tuberculous and scrofulous cheesy matter. Schüppel had found miliary tubercles in the caseous, so-called scrofulous lymph glands. The latter sometimes suppurate and periglandular abscesses form; these as well as the chronic, cold or scrofulous abscesses, contain in the lining membrane of the wall thousands of miliary tubercles. (Volkmann.) Scrofulous ulcers of the skin so common in children, grave cases of scrofulous ozæna, a number of anal fistulæ, obstinate ulcers of the soft palate and the pharynx in children and young individuals, where the malignancy of the ulcers was attributed to hereditary syphilis or their lupoid character, show, according to Volkmann's investigations, that miliary tubercles are the cause of their local destructive tendency.

The identity of scrofulosis and tuberculosis was slowly approaching the condition of an established fact. The above named investigations caused Schüller to enter the experimental field relating to fungous inflammations of the joints.

Rabbits and dogs were infected with tuberculosis, viz., scrofulosis in the following ways:

*a*. Tuberculous sputa from consumptive patients, or an emulsion of cheesy matter and miliary tuberculous tissue from human lungs was injected by means of an hypodermic syringe, the point of which was pushed through the thoracic walls.

*b*. The same kind of emulsion was injected into the trachea through the wound of a preceding tracheotomy.

*c*. By means of an atomizer the same emulsion was thrown into a closed space containing animals.

Besides the emulsion of tuberculous sputa and caseous and tuberculous tissue from the lungs, the author used for the infection of the animals a fluid containing secondary generations of micrococci, contained in the above-named material. The cultivation of these micrococci was produced in the following way: Miliary tuberculous lung tissue or cheesy matter from scrofulous glands was ground in a mortar in Bergmann's fluid for cultivating bacteria, thus forming an emulsion. Bergmann's fluid consists of 100 cubic centimeters of distilled water; 10 grams of rock candy; 1 gram of tartrate of ammonia, and ½ gram of phosphate of potassium.

The emulsion is then filtered, and of this milky filtrate from one to three drops are put into cleansed glasses, containing Bergmann's fluid. The glass is kept at a temperature of 30° C., and after three to four days the fluid becomes cloudy or milky from generated micrococci, and the so-called first culture or generation is completed. One to three drops of this is mixed in another glass of Bergmann's fluid, and results in three to four days in the second culture or generation. In the same way a third generation is produced from the second.

The fluid contains a multitude of small, round bacteriæ in very rapid motion. At 450 diameters they are to be seen as small round points. At 800 to 1200 diameters, and colored with methyl-violet they can be distinctly seen as spheroidal bodies, isolated or in groups of two or three. The second generation contained only these bacteria in a fluid free from the cells or particles of the original tubercles or cheesy matter. This fluid was then used for injection into the lungs or for inhalation with the same effect as the original emulsion of tuberculous tissue and cheesy matter. Rabbits and dogs were used for the experiments. The dogs and the larger and more powerful rabbits tolerated the operation readily and the tracheotomy wound healed up without accident. The infected animals after some time would commence to lose weight and become emaciated, and in spite of their good appetite would die in the course of three to ten weeks from tuberculosis of the lungs and other internal organs.

Traumatic lesions were now produced upon the joints of the infected animals. In the great majority of them the result was a chronic fungous arthritis, a tumor albus, a pannous arthritis.

The synovial membrane became thickened and covered with granulations (these granulations will also cover the peripheral portions of the cartilages), the cartilages thickened and became opaque, vascular, and finally transformed into the same kind of granulation tissue as found on the synovial membrane. The epiphyses of the joint became thickened and osteo-porotic, but no central carious cavity filled with cheesy matter was found in them. Towards the surface, however, in the enlarged medullary spaces was found an infiltration of lymphoid cells and consequent fatty degeneration. In and around those spaces miliary tubercles were constantly found. In the granulation tissue, in and on the synovial membrane miliary tubercles were found now and then but fewer in number and less frequently than in the medullary osseous tissue. Thus it was proved that a fungous arthritis of tuberculous character, a tuberculous synovitis commencing in the synovial membrane, was the result of slight traumatisms in animals infected with the tuberculous poison.

Deciding experiments were made in which the same traumatisms to the joints of non-infected animals were produced, and were not followed by any such chronic inflammation. A slight serous effusion or even extravasation of blood in and around the joint became absorbed in a few days, leaving the joint as healthy and movable as before.

The above experiments, together with the observed clinical cases where no primary local, osteo-tuberculosis is found after operation, tend to justify the opinion that a *primary tuberculous synovitis exists, not as a rare and grave disease as Volkman believed, but as one of the common forms of fungous arthritis,* that we so frequently meet with, resulting from slight traumatisms to the joints in scrofulous, *i. e.*, tuberculous individuals—mostly in children.

This primary tuberculous synovitis can further be a local tuberculosis as well as the osteo-tuberculosis of the epiphyses, as we have seen in our case of excision of the knee-joint, where the recovery is complete and the patient is growing stronger and gaining in weight, and not exhibiting the faintest signs of tuberculosis in any other organs of the body.

The relative frequency of the primary and secondary tuberculous arthritis must remain an open question at present, and will have to be determined by further clinical observations.

The practical bearing of the question is this: If the tuberculous arthritis, as Volkmann believes, is almost always secondary to a primary osteo-tuberculosis, we might hope in a large number of cases to be able to destroy the disease by local treatment previous to to the extension of the disease to the joint. Successful cases of this kind are reported by Kocher and Volkmann, but up to a recent date their number was only small, and we must regard them as exceptional cases.

COURSE AND TERMINATIONS OF THE TUBERCULOUS ARTHRITIS.

The object of this paper being only, or mainly, to elucidate and demonstrate the pathology of tuberculosis of the joints, we will have to postpone the discussion of the details of the course, viz., symptoms, complications, etc., until a future occasion when symptoms, treatment and its results will be illustrated by authentic cases of the disease. In this paper, however, we shall only point out some of the main indications on general principles.

At present, we know not what proportion of the cases of tumor albus, white swellings, or fungous arthritides are complicated with or dependent upon a miliary tuberculosis of the affected joints. Future investigations will have to solve the question: Are tubercles present in every case of fungous arthritis, or, in other words, is every case of funguous arthritis a local tuberculosis; or, still further, does there exist two distinct classes of of fungous arthritis, one tuberculous, and the other non-tubercular? From a pathological point of view this question is of vital importance as far as the prognosis is concerned, because a non-tubercular arthritis would be a benignant disease, not for the joint, but for the life of the patient, as there would be no danger of a general tuberculosis resulting therefrom. The investigations of Volkmann and Friedländer during the last years seem to prove that the great majority of the fungous arthritides are of a true miliary tuberculous nature. If this be the true state of things we shall have to change our inherited views of the malignancy of the miliary tuberculosis, and we shall have to admit that a local tuberculosis of a bone or a joint is so far benignant that it may terminate in a relative recovery, viz., in anchylosis of the joint without the necessity of involving a fatal general tuberculosis of the lungs or any other system of organs necessary for the continuation of life.

It is now beyond doubt that a number of tuberculous arthritides terminate in what we used to call recovery, that is, a more or less complete fibrous or osseous anchylosis of the affected joint. It is further possible that this recovery may be a complete one as to the tuberculosis, viz., that all the tubercles in the fungous arthritis have disappeared, and the tissues of the anchylosis consist of fibrous and osseous tissue apt to persist for an indefinite period of years without any danger, either from future local inflammation or from general tuberculosis.

A very interesting point in Schüller's experiments we wish to mention in connection with this side of question. Consequent to the experiments with the bacteriæ developed in Bergmann's fluid, Schüller raised the question whether or not antidotes of anti-bacteriac remedies would not show a beneficial influence upon the tuberculous infected animals. He pretends to have found that inhalations of a five per cent. watery solution of benzoate of soda thrown into the respiratory tract by means of atomizers was effective, sometimes curing and always ameliorating the condition of the tuberculosed animals. Under this treatment they gained in weight, the tuberculous arthritis got better and, if not too far gone, disappeared entirely.

How common this complete recovery is we do not know at present. But we shall here point out and demonstrate the fact that the recovery from a tuberculous arthritis may seem complete for years and still hide the germ of a finally fatal tuberculosis.

Fig. 7 shows an anchylosed hip joint, removed at a post mortem examination of a man about thirty-five years old, who died in the Cook County Hospital from chronic pulmonary tuberculosis, *i. e.*, consumption. The patient, when about fifteen years old, had suffered from morbus coxarius which had terminated in an anchylosis and left him a relatively useful limb, on which he was able, in spite of the false position—flexion almost to a right angle with the perpendicular line of the body—to walk or limp around for a good many years. We find, as is shown in the figure, a tuberculous abscess filled with cheesy mater, situated on the pelvic surface of the corpus ossis ischii.

This abscess communicates with two osteo-tuberculous cavities; the upper one in the acetabular portion of the os ischii, the other in the atrophic head of the anchylosed os femoris. Still lower in the head of the femur is another small, round, tuberculous cavity (13) that has no communication with the above described ones. This local tuberculosis of the hip joint and its surrounding bones was the primary disease. The arthritis terminated in an anchylosis. The tuberculous cavities, filled with cheesy matter and lined with a membrane containing thousands of miliary tubercles, remained for a long series of years harmless, but finally were the source of an infection from which emanated the fatal tuberculosis of the lungs.

The tuberculous cavities in the bones, in these cases, and the tuberculous abscesses around the bones keep the patient in constant danger of a general tuberculosis in the vital organs of the body. How long a period—how many years the local tuberculosis will remain latent we do not know. Neither do we know the circumstances or causes under which the tuberculous poison from those hitherto quiet foci spreads out from its local seat into the organism and causes either an acute miliary tuberculous meningitis of the base of the brain, or an acute miliary tuberculosis of the lungs, or a chronic tuberculosis of lungs, genito-urinary organs, peritoneum, etc.

We now very naturally have to ask the question: Will this new light thrown upon the cause and nature of fungous arthritis have any influence on the treatment? Will we have in the future to operate on cases in which we before trusted to an expectant and less bloody means of cure? We must answer the question in the affirmative, but at the same time warn against an unlimited cutting away, regardless of results, of every particle of tuberculous tissue of the local tuberculosis, in view of avoiding a fatal generalization as unnecessary and not indicated. It certainly was for many years a *pium desideratum* to be able to cut away old cheesy deposits in lungs and bronchial glands, as the pathologist saw in numerous cases the deadly general tuberculosis start out from these very places. In later years this *desideratum*, to prevent general infection, was partly realized by removal* of cheesy glands from the neck, and scraping out of scrofulous ulcers with the sharp spoon. But as we learned to know the true tuberculous character of a great many of the so-called scrofulous inflammations, cheesy glands, sores and fistulæ, some varieties of lupus, we were obliged to give up part of our inherited dread of miliary tubercles, knowing that a number of the last named local tuberculoses heal up without operation and without being followed by fatal general tuberculosis.

The still considerable number of cases where the local tuberculosis leads to a fatal generalization compels us to operate where

---

* Hüter: Die Scrofulosis und ihre locale Behandlung als Profylaxe gegenüber der Tuberculose. Volkmann Klinische Vorträge Chirurgie No. 15.

the removal can be effected without danger to the life or any considerable destruction of the part affected.

The frequency of a primary osteo-tuberculosis, as the cause of the fungous arthritis, demands a minute and constant watching of the initial stage of the disease, and as soon as we may be able to make the diagnosis of the affected locality of the bone, we will have to proceed to its removal by gouge, trephine and currette, etc., or its destruction by red hot iron (Kocher.) It is completely useless in these cases to lose time by relying on the application of external remedies, even down to actual cauterization, though the whole thickness of skin and subcutaneous tissue be destroyed by the same. They will have no effect at all upon the local tuberculosis in the interior of the bone, and this will proceed undisturbed on its way toward the destruction of the joint.

Further, we shall have to make a change in the old indications for resections—excisions—of the joints. The majority of surgeons have been used to make the excisions late, often too late, in the course of the disease. We were accustomed to require discharging fistulæ, or palpable periarticular abscesses, besides crepitation on motion of the joint, which latter means a carious destruction of the articular surfaces. Now we find in a number of cases of extensive tuberculous destruction of the epiphyses and fungous destruction of the synovial membrane, no fistulæ, no abscesses, no crepitation on moving the joint, and nevertheless the excision strongly indicated, as shown by the records of the knee case, (Fig. 4) which we have before mentioned. In a case at Cook County Hospital, where a successful excision of the knee joint was performed by Dr. Isham, there were no fistulæ, no abscesses, and only slight swelling of the joint.

The boy had for years been unable to use the limb, on account of pain in the joint. There was some angular deviation outwards and some lateral mobility owing to destruction of the internal lateral ligament. The exsection revealed, besides a fungous arthritis with partial destruction of the articular cartilaginous surfaces, a tuberculous cavity filled with cheesy matter in the upper epyphysis of the tibia, extending down below the cut surface of the latter. The exsection combined with *évidement* of the lower part of the cavity, and drainage through a drilled opening through the anterior surface of the epiphysis, affected a complete recovery viz., an anchylosis with a useful limb.

As we have already pointed out, we must leave the details of indications for operation, and details of symptoms and course for future clinical records of cases, which will be published as a continuation of our present paper. We shall here only mention the two main points for consideration, before we resort to excision of a joint.

The first is, we require as the probable result of the operation, a more useful limb for the patient than he had before the excision, or probably would have within a reasonable time without surgical interference.

The second ; we insist and expect, in removing the tuberculous joint to free the patient from an ever-existing source for general tuberculosis—a sword of Damocles hanging over and constantly menacing his very life.

The larger the quantity of cheesy matter, the less the possibility of a speedy absorption of this infectious substance, the greater will be the importance of the second point as an indication for operative interference.

But the whole modern tendency towards conservatism in surgery demands that the question of general tuberculosis be put second to the question of the function of the joint or the limb. In deciding otherwise we would run a risk for an uncertain gain, the percentage of which is as yet not known, in operating on cases where non-interference would give as good results with less, or without temporary danger to the life of the patient. Future observations will have to determine the relative weight of these two main factors in the indications for operative interference.

It will only be through numerous carefully-observed cases that future surgery will be able to decide upon the best course of procedure in each single instance of the disease in question.

We would be glad to hope that our paper of to-night may bring numbers of the profession here to participate in the further development of our knowledge to, and in the solution of the main questions regarding the miliary tuberculosis of the joints.

## Figures and Tables

**Fig. 1. f1:**
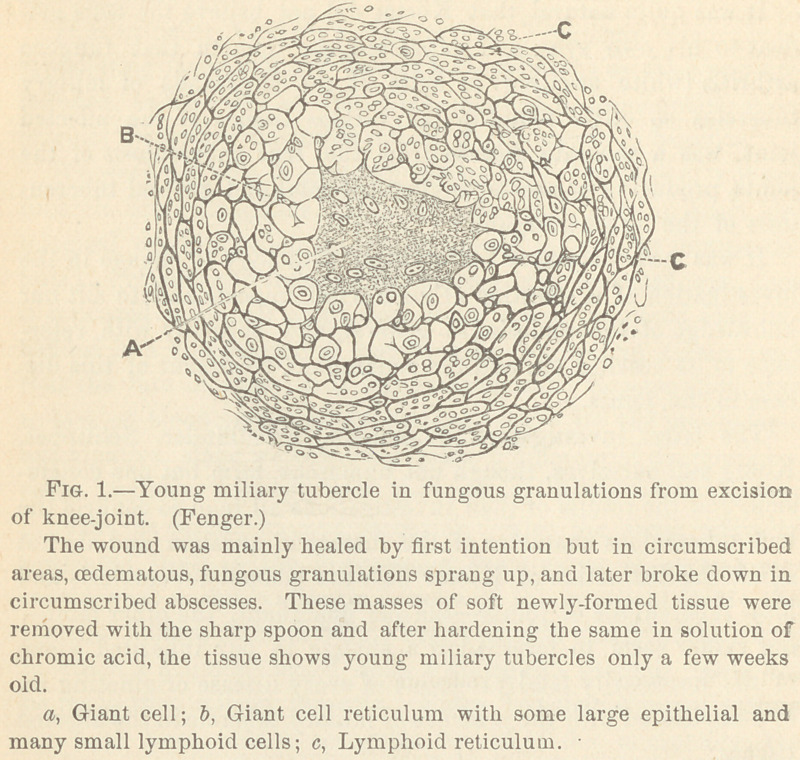


**Fig. 2. f2:**
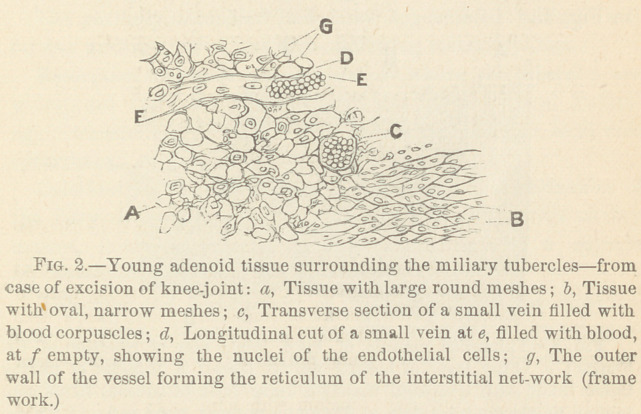


**Fig. 3. f3:**
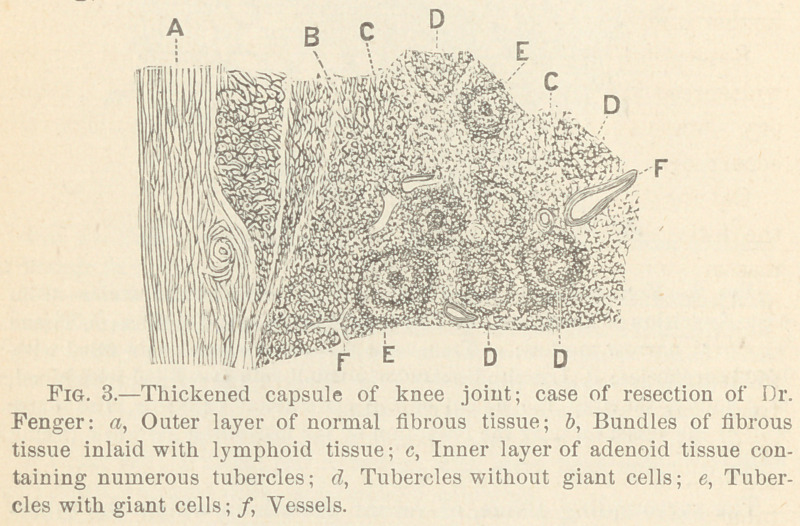


**Fig. 4. f4:**
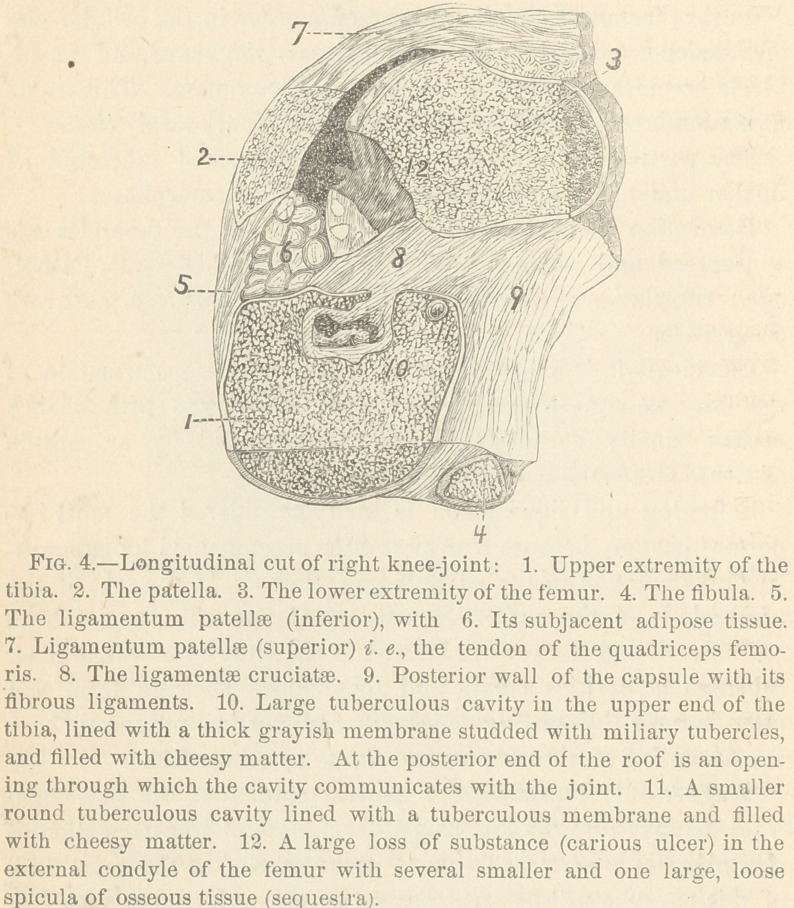


**Fig. 5. f5:**
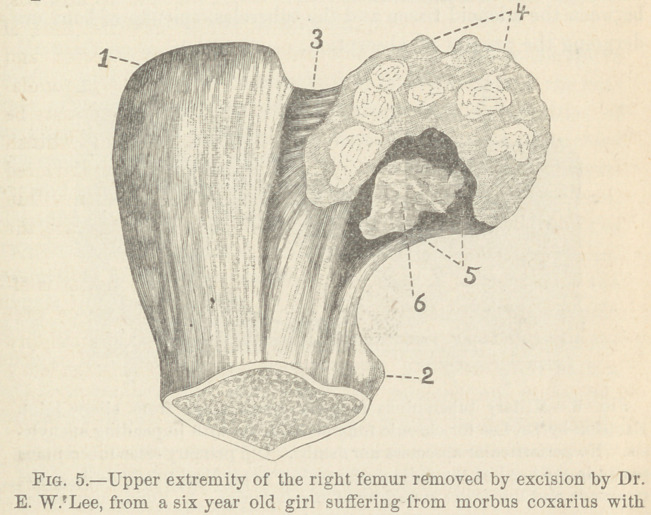


**Fig. 6. f6:**
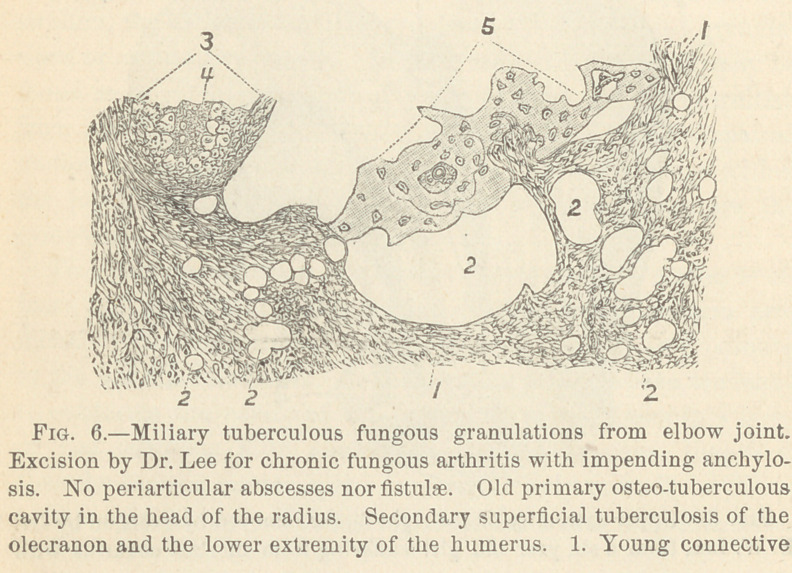


**Fig. 7. f7:**